# Classroom and family ecology of academic achievement emotions: a cross-cultural comparison between China and Kazakhstan

**DOI:** 10.3389/fpsyg.2026.1922663

**Published:** 2026-09-17

**Authors:** Zhan Zhou, Aigul Alchimbayeva, Chunling Wang, Xiaogang Jin

**Affiliations:** 1 Faculty of Philosophy and Political Science, Al-Farabi Kazakh National University, Almaty, Kazakhstan; 2 Faculty of Arts, Huanggang Normal University, Huanggang, China

**Keywords:** academic emotions, classroom goal climate, cognitive appraisals, cross-cultural comparison, life satisfaction, parental expectations and support

## Abstract

**Introduction:**

Guided by a core mediation model (environment → cognitive appraisals → emotions → life satisfaction), this study explores the multiple structural associations among classroom goal climate, perceived parental academic expectations and support (PPASS), cognitive appraisals, academic emotions, and life satisfaction across different cultural contexts.

**Methods:**

A cross-sectional survey was conducted among 1,645 university students from China and Kazakhstan. Multi-group confirmatory factor analysis (MGCFA) was utilized to verify the measurement invariance of the instruments, and multi-group structural equation modeling (MG-SEM) was employed to analyze the structural associations across the two groups.

**Results:**

After confirming strong measurement invariance across both groups, the MG-SEM revealed that mastery climate was significantly and positively associated with perceived control and task value. Perceived control covaried positively with positive emotions. The path from task value to positive emotions was not significant in either sample, while task value and negative emotions were significantly and negatively associated in both samples. Parental expectations and support were positively associated with task value, and indirectly linked to lower negative emotions via the task value pathway. Notably, the negative covariation between perceived control and negative emotions was significant only in the Kazakhstani sample. Furthermore, positive and negative emotions were significantly positively and negatively associated with life satisfaction, respectively.

**Discussion:**

These findings theoretically elucidate the cross-cultural psychological mechanisms of educational ecologies, suggesting that higher education interventions should foster mastery-oriented environments and autonomy-supportive family involvement to facilitate students' psychological adaptation.

## 1 Introduction

### 1.1 Background and problem statement

The internationalization of contemporary higher education presents differentiated evolutionary paths across various geopolitical and cultural contexts. Since its independence, Kazakhstan, located in Central Asia, has continually advanced the institutional transition of its higher education to align with the European Bologna Process, requiring students to establish a new psychological balance between traditional family values and modern academic demands (Abdigapbarova et al., 2025; Winter et al., 2022). In China, under the “Double First-Class” university construction initiative and a highly selective social screening mechanism, university students face high academic pressure. Against these two educational backgrounds, the psychological adaptation of university students has become a shared issue of concern in the higher education sectors of both China and Kazakhstan. Psychological adaptation during the university stage is largely reflected through academic emotions. Existing cross-cultural surveys show that despite the continuous improvement in higher education levels, the detection rates of negative emotions such as anxiety, shame, boredom, and anger remain high among university students (Li, 2021). Particularly after undergoing major academic assessments, Chinese students are highly susceptible to deep emotional fluctuations such as pride, shame, and self-denial induced by external comparisons and evaluations (Fang et al., 2023). These emotional fluctuations are not only related to learning outcomes but are also linked to individuals' evaluations of life satisfaction. However, existing studies have mostly been confined to single campus environments, failing to integrate macro-cultural systems, family and school microsystems, and their mesosystem connections into a unified analytical framework.

### 1.2 Theoretical framework: ecological systems theory and control-value theory

To systematically present the relational structure between environmental factors and individual emotional experiences, this study integrates Bronfenbrenner's (1979) bioecological systems theory with Pekrun's (2006) control-value theory (CVT). CVT posits that the environment in which an individual is situated is linked to various academic emotions mediated by two types of cognitive appraisals: “perceived control” and “perceived value” (Pekrun et al., 2007).

Under the ecological systems theory framework, an individual is embedded in an educational ecology composed of multiple microsystems and the mesosystem formed by interactions between these microsystems. Among them, the classroom goal climate (belonging to the school microsystem) and parental expectations and academic support (PPASS, belonging to the family microsystem) jointly constitute the external environmental background for students' cognitive appraisals, and their synergistic effect reflects joint shaping at the mesosystem level. Furthermore, macro-cultural values outside the microsystems (such as collectivism and power distance) moderate the relational strength when these environmental pressures are transmitted to individual cognition and emotion. Integrating macro-system cultural values and the family and classroom microsystems that embody mesosystem connections into a unified analytical framework helps to more comprehensively understand the structural mechanisms of university students' emotional adaptation. Specifically, within this integrated framework, the key variables are operationalized along an ecological structure: classroom goal climate acts as the school microsystem variable reflecting teaching orientations, while parental expectations and support conceptually capture the family microsystem's dual nature of pressure and resource supply. At the individual level, cognitive appraisals (perceived control and task value) function as the internal evaluative mechanisms bridging these external environments. Furthermore, academic emotions map the immediate psychological adaptations that are systematically associated with an individual's global life satisfaction.

### 1.3 Research objectives

Based on the theoretical framework above, this study conducts an empirical cross-cultural comparison to examine the structural associations among classroom climate perceptions, family ecology perceptions, control-value cognitive appraisals, academic emotions, and overall life satisfaction of university students in China and Kazakhstan.

The higher education ecology in Kazakhstan integrates the collective mutual assistance traditions of nomadic culture, the educational system legacy of the Soviet era, and the influence of diversified values post-independence; Chinese surveyed students, on the other hand, are situated in a cultural background characterized by Confucian family-oriented values and strong socially-oriented achievement motivation. Conducting isomorphic measurements and comparisons of these two groups can provide cross-cultural empirical evidence for the psychological adaptation mechanisms in higher education. By doing so, this study contributes both theoretically and practically. Theoretically, it synthesizes CVT and Ecological Systems Theory (EST) to construct a progressive logical chain showcasing how macro-cultural values and micro-environmental systems are structurally related to individual emotional adaptation. Practically, the findings offer empirical guidance for educators and mental health practitioners to design culturally sensitive psychological interventions and pedagogical strategies in diverse higher education contexts.

## 2 Literature review & hypotheses

### 2.1 Association between classroom goal climate (PALS) and academic achievement emotions

Micro-teaching contexts constitute an important external environmental source for students' emotional experiences. According to the definition by Midgley et al. (2000), classroom goal structures are mainly divided into the mastery climate, which emphasizes knowledge understanding and cognitive improvement; the performance-approach climate, which focuses on performance and competitive ranking; and the performance-avoidance climate, which aims to avoid exposing one's shortcomings. In CVT, different goal climates transmit different evaluative feedback signals to individuals. When teachers emphasize individual growth and self-transcendence (mastery climate), students are more likely to perceive a sense of control over learning tasks, showing a stronger positive association with positive emotions such as enjoyment and hope (Baudoin and Galand, 2022; Datu et al., 2022). If there is a frequent ranking atmosphere within the class and failing is viewed as incompetence (performance-avoidance climate), individuals' attention usually shifts from learning content to worrying about the consequences of failure, showing a stronger positive association with negative emotions such as anxiety and shame (Fejes, 2023; Goetz et al., 2008). In addition, the performance-approach climate, which focuses on demonstrating personal ability and outperforming others, exhibits a more complex dual attribute regarding the association between cognitive appraisals and emotions. Under this climate, the highly competitive atmosphere may, on the one hand, combine with students' need to prove their abilities, showing positive covariation with positive indicators like task value and pride; on the other hand, excessive focus on relative ranking may also be associated with reduced perceived control, subsequently showing positive covariation with negative emotions like anxiety (this is also related to teachers' micro-judgments of student achievement: Zhu et al., 2018). Based on the above analysis, the following hypotheses are proposed:

H1a: Classroom mastery climate (MAS) is positively associated with positive academic emotions (Enjoyment, Hope, Pride).
H1b: Classroom performance-avoidance climate (PAV) is positively associated with negative academic emotions (Anxiety, Shame, Boredom, Anger).
H1c: Classroom performance-approach climate is significantly associated with university students' perceived control and academic emotions.

### 2.2 Association between family microsystem environment (PPASS) and individual perceived control

The family has a crucial impact on an individual's psychological development. The latest longitudinal and cross-cultural evidence also indicates that parental academic expectations and aspirations can significantly predict and shape students' academic achievement emotions (Sakaki et al., 2026). In the research framework of Marchant et al. (2001), parental academic expectations and support across different dimensions will bring different psychological perceptions to individuals in the autonomy development phase of the university stage. PPASS, as a composite construct, includes both high parental academic expectations and daily performance monitoring (which tends to compress individual autonomy and is interpreted by the individual as control), as well as active help and support when facing academic difficulties. The development of this family ecology perception has a certain complexity; on the one hand, high expectations and monitoring may compress an individual's perceived control; on the other hand, active resource support helps build the individual's efficacy. Meanwhile, the gap or divergence in educational expectations and aspirations between parents and children is often closely related to the individual's psychological wellbeing and daily school adaptation levels (Zeng et al., 2024). Therefore, drawing upon traditional perspectives of autonomy development, the following hypotheses are proposed: H2a: Due to the potential suppression of autonomy by expectation monitoring, perceived parental expectations and support (PPASS) is negatively associated with university students' perceived control, but positively associated with task value. H2b: Perceived parental expectations and support have an indirect association with lower levels of negative academic emotions through the mediating mechanism of task value.

### 2.3 Mediating role of cognitive appraisals: perceived control and task value (CVA)

CVT asserts that external environments (e.g., classroom goal climate, parental behavior) do not directly trigger an individual's academic achievement emotions but are mediated through the individual's control appraisals and value appraisals (Pekrun, 2006; Tze et al., 2023). Specifically, this study constructs a progressive logical chain wherein CVT's cognitive appraisal mechanisms operationalize the broader structural associations of EST's school and family microsystems. In this integrated model, CVT serves as the internal psychological framework that structurally links distal ecological environments to proximal emotional responses. Perceived control represents an individual's efficacy estimation of whether they can achieve academic goals through effort, while perceived value points to the individual's judgment of the importance of academic activities in instrumental or intrinsic attributes (Pekrun et al., 2002). When the external environment (such as a mastery climate) implies that tasks can be overcome through effort, an individual's sense of control and value cognition increase accordingly, providing a basis for positive covariation with emotions such as enjoyment and pride; if parental pressure or avoidant classroom rules imply a high probability of failure, perceived control levels decrease, accompanied by higher anxiety (Li and Li, 2024; Li et al., 2025). From a longitudinal dynamic perspective, the fluctuating development of an individual's control and value appraisals can effectively predict changes in their positive emotions, such as learning enjoyment (Hawrot, 2024). Therefore, the following hypothesis is proposed:

H3: Perceived control and perceived value play a mediating role in the association between parental expectations and support, classroom climate, and university students' academic achievement emotions.

### 2.4 Association between academic emotions and life satisfaction (SWLS)

Achievement emotions possess a spillover characteristic that extends beyond the academic context. The university stage is a critical period for young individuals transitioning to adulthood, and academic experience is an important component of an individual's evaluation of life satisfaction. Positive academic emotions (such as hope) can often generate positive experiences in daily life, leading students to give higher evaluations of their life state. Continuously accumulated negative emotions (such as test anxiety, shame, boredom, and anger) are associated with lower overall life satisfaction (Lu et al., 2021; Zhou et al., 2024). This transformation mechanism from academic motivation and emotional experience to individual life satisfaction constitutes the core closed-loop of students' school life adaptation and wellbeing (Feraco et al., 2023). Therefore, the following hypothesis is proposed:

H4: Positive academic achievement emotions are positively associated with overall life satisfaction, whereas negative academic achievement emotions are negatively associated with overall life satisfaction.

### 2.5 Moderating role of cultural values (CVS)

Cultural values are an important background variable for individuals to understand the environment and construct a meaning framework (Hofstede, 1984). In the classification of cultural values by Dorfman and Howell (1988), collectivism and power distance have strong structural impacts in East Asian and Central Asian societies. To better contextualize these variables, it is necessary to differentiate the macro-cultural backgrounds of China and Kazakhstan. In Chinese Confucian culture, authority, status, and rank within families and schools are deeply respected, rendering high power distance and family-oriented collectivism normative. Under this background, strict parental expectations and control are often intertwined with filial piety and interpreted as legitimate familial care (Lin and Wang, 2022). Conversely, Kazakhstan's macro-cultural values blend the nomadic heritage of traditional mutual assistance with modern educational trends. Within this transitional context, power distance is less absolute, and traditional values gradually shift toward community solidarity rather than strict hierarchical family compliance (Toleubekova et al., 2022).

Collectivism here manifests as a family-collective orientation (i.e., academic achievement is linked to family honor and parental expectations). Among students who highly identify with family-style collectivism, parental academic expectations and support may be internalized as the individual's internal driving force, thereby alleviating their feelings of lack of control; however, when encountering academic setbacks, a high collectivist orientation may be associated with stronger feelings of shame due to thoughts of “failing the family” (Tan et al., 2021). Furthermore, regarding power distance, it may exhibit a culturally specific moderating capacity: in high power distance contexts, authoritative parental expectations might be accepted as customary, showing a weaker statistical association with negative emotional resistance; whereas in lower power distance contexts, identical expectations might be perceived as oppressive, demonstrating a stronger positive correlation with perceived stress and negative emotions (Acoba, 2024).

Nevertheless, due to individuation and separative development during the university stage, collectivism might also blunt this immediate pressure fluctuation. Based on this, the present study examines whether collectivism and power distance play a moderating protective role, proposing the following hypothesis:

H5: Cultural values, represented by individual collectivism and power distance, play a moderating role in the association between parental expectations and support and negative academic emotions across different cultural orientations.

Thus far, focusing on the relational mechanisms among the external environment, cognitive appraisals, academic emotions, and life satisfaction, this study has constructed a complete research hypothesis path network, as shown in Figure 1.

**Figure 1:**
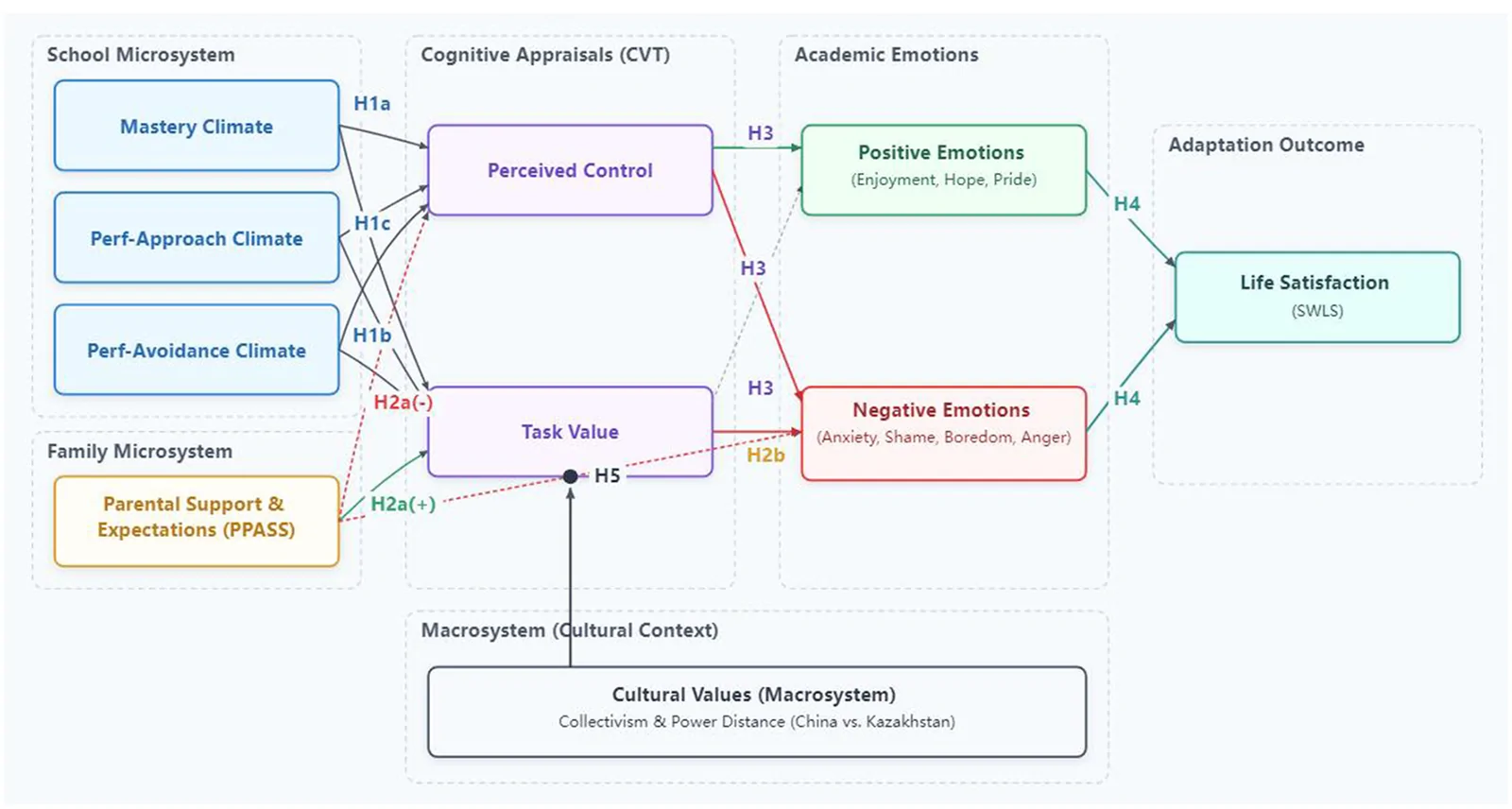
The path relationship diagram of research hypotheses proposed in this study.

## 3 Methodology

### 3.1 Participants and procedures

This study employed a cross-sectional survey method, utilizing convenience sampling to select current university students from universities in Shanghai (and its surrounding regions), China, and the Almaty region, Kazakhstan, as survey subjects. Selecting these two samples aimed to contrast the highly selective and competitive higher education ecology in East Asia with the transitional mixed higher education ecology in Central Asia, providing a cross-cultural comparison platform to examine the psychological covariation characteristics of students under different educational backgrounds.

Data collection was completed between March and May 2025 via an online survey platform. The presentation format of the questionnaire, response instructions, and anonymity statements remained consistent in both countries. After obtaining informed consent from the relevant university administrative departments and the students, the Chinese sample was distributed via targeted online links; the Kazakhstani sample was recruited with the help of local research assistants utilizing local university online communities.

The online system collected an initial total of 1,760 response records (900 in the Chinese group and 860 in the Kazakhstani group). In the data cleaning stage, invalid questionnaires with excessively short completion times (< 120 s) or obvious regular response patterns were first excluded, yielding 1,645 valid questionnaires. Subsequently, Mahalanobis Distance (*D*^2^) was utilized to identify and remove multivariate extreme outliers. The final analytical sample consisted of 865 surveyed students from China and 780 surveyed students from Kazakhstan. The basic demographic characteristics and distributions of the overall sample are shown in Table 1.

**Table 1:** Distribution of basic demographic characteristics in the two country samples.

Demographic characteristics	Category	China (*n* = 865)	Kazakhstan (*n* = 780)	Total (*N* = 1,645)
Gender structure	Female	586 (67.7%)	434 (55.6%)	1,020 (62.0%)
	Male	266 (30.8%)	308 (39.5%)	574 (34.9%)
	Other/refused to answer	13 (1.5%)	38 (4.9%)	51 (3.1%)
Age distribution	18–20 years old	361 (41.7%)	403 (51.7%)	764 (46.4%)
	21–23 years old	394 (45.6%)	314 (40.3%)	708 (43.0%)
	24 years old and above	110 (12.7%)	63 (8.1%)	173 (10.5%)
Study stage	Undergraduate	618 (71.4%)	658 (84.4%)	1,276 (77.6%)
	Postgraduate (master's & doctoral)	237 (27.4%)	114 (14.6%)	351 (21.3%)
	Other stages	10 (1.2%)	8 (1.0%)	18 (1.1%)
Family background	Parents' highest education ≥ bachelor's	488 (56.4%)	398 (51.0%)	886 (53.9%)

“Other stages” in the study stage category primarily refers to non-degree visiting scholars, exchange students, and continuing education students at the university level.

### 3.2 Measures and translation equivalence

All original English psychological measurement instruments were processed using standard “Translation and Back-translation” procedures. Two bilingual scholars with a background in psychological research translated the English scales into Chinese and Russian, respectively, and then two independent translators completed the back-translation into English. The project team conducted semantic comparisons and corrections on the back-translated versions, making localized adjustments to vocabulary that might generate different understandings in Chinese and Russian contexts to ensure the measurement constructs possessed equivalent semantic connotations across both country samples (detailed questionnaire sets and codebooks are available in the Supplementary material). The survey questionnaire in this study contained a total of 60 measurement items. Before formal data analysis and invariance testing, reverse-scoring formula conversions were applied to the reverse-worded items included (CVS_COL_04_R and CVA_CTR_03_R), and all 60 core scale items participated in common method bias and configural invariance analyses to ensure the completeness and unbiasedness of statistical estimations.

Academic Emotions (Achievement Emotions): Adopted the simplified version of the Achievement Emotions Questionnaire (AEQ) developed by Pekrun et al. (2011). The scale contains seven emotional dimensions: Enjoyment (three items), Hope (three items), Pride (three items), Anxiety (four items), Shame (three items), Boredom (three items), and Anger (three items), totaling 22 items, evaluated on a 5-point Likert scale (1 = strongly disagree, 5 = strongly agree).

Classroom Goal Climate (PALS): Excerpted from the Patterns of Adaptive Learning Scales (PALS; Midgley et al., 2000), including three dimensions: Mastery-oriented Climate (four items), Performance-oriented Climate (four items), and Performance-Avoidance Climate (three items), totaling 11 items, scored on a 5-point scale.

Parental Expectations and Academic Support (PPASS): Adapted from the Perceived Parental Academic Support Scale (PPASS) developed by Marchant et al. (2001), containing five items. This scale assesses the individual's perceived parental academic achievement expectation goals, frequency of performance monitoring, parental disappointment reactions when performance targets are not met (four items in total), as well as active assistance and support provided by parents when facing academic difficulties (one item), scored on a 5-point Likert scale. Higher scores indicate stronger perceived parental academic expectations and active support.

Control-Value Appraisals (CVA): Designed according to the appraisal constructs of the control-value theory (Pekrun et al., 2007), including Perceived Control (four items, of which item 3 is reverse-scored) and Perceived Value (four items), scored on a 5-point scale.

Cultural Values (CVS): Selected the dimensions of Collectivism (four items, of which item 4 is reverse-scored), Power Distance (three items), and Uncertainty Avoidance (two items) from the Cultural Values Scale developed by Dorfman and Howell (1988), all scored on a 5-point Likert scale.

Global Life Satisfaction (SWLS): Used the Satisfaction With Life Scale (SWLS) by Diener et al. (1985), containing five items, evaluated on a 7-point Likert scale (1 = strongly disagree, 7 = strongly agree).

### 3.3 Statistical analysis strategy

This study used SPSS 26.0 (IBM Corp., Armonk, NY, USA) and the semopy library in Python to conduct statistical analysis, proceeding in the following three stages:

Pre-data processing and variable extraction: To ensure the convergence of the macro-structural model and theoretical parsimony, this study employed structural dimension synthesis for dimensionality reduction of the measurement data before formal modeling analysis. Specifically, the mean values of the three sub-dimensions of enjoyment, hope, and pride were synthesized into a comprehensive index of positive academic emotions (AEQ_POS), and anxiety, shame, boredom, and anger were synthesized into a comprehensive index of negative academic emotions (AEQ_NEG) for unified analysis. In addition, the Uncertainty Avoidance (CVS_UAV) dimension in the cultural values scale was not included in the path system in subsequent multi-group structural equation models and mediation effect tests due to its extremely low zero-order correlation coefficients with the main dependent variables in the pre-analysis, adhering to the principle of structural equation path parsimony.

Measurement Invariance Testing: To ensure that cross-group comparisons are based on the same measurement structure, this study designed and ran nested comparison tests of Multi-Group Confirmatory Factor Analysis (MGCFA): sequentially testing Configural Invariance, Metric Invariance (constraining factor loadings to be equal across groups), and Scalar Invariance (constraining item intercepts to be equal across groups). The difference criteria for model nesting iteration were set as Δ Comparative Fit Index (CFI) ≤ 0.01 and Δ Root Mean Square Error of Approximation (RMSEA) ≤ 0.015.

Structural Equation Path Modeling and Difference Testing: Constructed structural equation path models containing aggregated observation variable sets to examine the relational structures among environment, emotional appraisals, and life satisfaction. Mediation effect analysis employed bias-corrected non-parametric percentile Bootstrap techniques (5,000 resamples) and derived 95% confidence intervals based on Hayes (2018); multi-group structural equation modeling was used to verify cross-group path differences (relying on Wald χ^2^ difference estimations). Cohen's *d* and *f* 2 were introduced in the mean comparisons and moderation effect analyses to quantify effect sizes, respectively.

## 4 Results

### 4.1 Data diagnostics and common method bias testing

Collinearity diagnostics were performed on each predictor variable in the model. The Variance Inflation Factors (VIF) ranged from 1.048 to 1.148 in the Chinese sample and 1.053–1.164 in the Kazakhstani sample, all significantly lower than 3.0, ruling out severe collinearity risks. The common method bias test results showed that the maximum variance explained by Harman's single factor was 9.19 and 9.10% in the Chinese and Kazakhstani samples, respectively, far below 40%. After introducing the Unmeasured Latent Method Construct (ULMC) model for nested comparison, the changes in ΔCFI for both groups of models were < 0.010, indicating that common method bias did not constitute a substantial threat to path estimation (see Table 2).

**Table 2:** Diagnostics results for multicollinearity and common method bias.

Diagnostic index	Threshold	China sample (*n* = 865)	Kazakhstan sample (*n* = 780)	Diagnostic conclusion
VIF (predictor/mediating variables)	< 3.0	1.048–1.148	1.053–1.164	No collinearity issues
Harman's single factor max variance explained	< 40%	9.19%	9.10%	No single factor dominance
ULMC nested model ΔCFI	≤ 0.050	< 0.010	< 0.010	Method bias impact negligible

### 4.2 Reliability and confirmatory factor analysis validity testing

To assess the psychometric quality of the measurement instruments in the samples from China and Kazakhstan, this study calculated the internal consistency reliability coefficients (Cronbach's α), Composite Reliability (CR), and Average Variance Extracted (AVE) for each latent variable, as shown in Table 3.

**Table 3:** Reliability and validity test results of latent variables in the China and Kazakhstan samples.

Latent variable	China α	China *AVE*	China *CR*	Kazakhstan α	Kazakhstan *AVE*	Kazakhstan *CR*
PALS_MAS (mastery climate)	0.737	0.559	0.835	0.747	0.569	0.840
PALS_PER (performance-approach climate)	0.715	0.540	0.824	0.743	0.566	0.839
PALS_PAV (performance-avoidance climate)	0.785	0.700	0.875	0.762	0.678	0.863
PPASS (parental expectations and support)	0.790	0.544	0.856	0.741	0.491	0.828
CVA_CTR (perceived control)	0.725	0.549	0.829	0.702	0.531	0.817
CVA_VAL (task value)	0.741	0.564	0.838	0.747	0.568	0.840
AEQ_POS (positive academic emotions)	0.853	0.582	0.887	0.844	0.554	0.869
AEQ_NEG (negative academic emotions)	0.881	0.532	0.893	0.862	0.505	0.871
SWLS (life satisfaction)	0.763	0.515	0.841	0.729	0.482	0.822
CVS_COL (collectivism)	0.737	0.562	0.836	0.703	0.534	0.819
CVS_PWD (power distance)	0.758	0.674	0.861	0.750	0.667	0.857
CVS_UAV (uncertainty avoidance)	0.717	0.779	0.876	0.831	0.855	0.922

As shown in Table 3, the Cronbach's α for all latent variables in both the Chinese and Kazakhstani samples was above 0.70, and CR was above 0.81, indicating that each scale has good internal consistency reliability and composite reliability. Except for the average variance extracted (AVE) of parental expectations and support (PPASS, 0.491) and life satisfaction (SWLS, 0.482) in the Kazakhstani sample, which were slightly lower than the strict threshold of 0.50, the AVEs of all other latent variables were >0.50. Given that the composite reliability (CR) of these latent variables was already >0.80, according to the academic recommendations of Fornell and Larcker (1981), when CR meets the standard, a slightly lower AVE can still be judged as having convergent validity within an acceptable range.

Discriminant validity was tested using the Fornell–Larcker criterion (see Tables 4, 5). In this test, the academic emotion sub-dimensions were replaced by the two higher-order latent constructs actually used in the main research model: positive academic emotions (AEQ_POS) and negative academic emotions (AEQ_NEG), and the uncertainty avoidance (CVS_UAV) dimension was included. The results showed that the square root of the AVE for each latent variable was greater than the absolute values of its Pearson correlation coefficients with other latent variables, indicating good discriminant validity among the constructs.

**Table 4:** Discriminant validity test matrix for the China sample (Fornell–Larcker criterion).

Latent variable	(1)	(2)	(3)	(4)	(5)	(6)	(7)	(8)	(9)	(10)	(11)	(12)
(1) PALS_MAS	**0.747**											
(2) PALS_PER	0.009	**0.735**										
(3) PALS_PAV	0.043	0.315	**0.837**									
(4) PPASS	−0.027	0.045	0.067	**0.738**								
(5) CVA_CTR	0.226	0.033	0.083	0.014	**0.741**							
(6) CVA_VAL	0.240	−0.014	0.045	0.188	0.234	**0.751**						
(7) AEQ_POS	0.252	0.065	0.027	−0.011	0.240	0.015	**0.763**					
(8) AEQ_NEG	−0.008	0.148	0.217	0.034	−0.015	−0.149	−0.072	**0.729**				
(9) CVS_COL	0.101	0.028	−0.012	0.248	−0.006	0.045	−0.045	−0.023	**0.750**			
(10) CVS_PWD	0.059	−0.019	0.233	−0.044	0.045	0.052	0.024	−0.005	−0.034	**0.821**		
(11) SWLS	0.191	−0.011	0.007	0.013	0.254	0.260	0.382	−0.354	0.003	0.070	**0.718**	
(12) CVS_UAV	−0.042	0.060	0.066	0.022	−0.010	0.008	−0.049	0.017	−0.019	−0.038	0.017	**0.883**

Bold values on the diagonal are the square roots of AVEs for each latent variable; the remaining values are Pearson correlation coefficients.

**Table 5:** Discriminant validity test matrix for the Kazakhstan sample (Fornell–Larcker criterion).

Latent variable	(1)	(2)	(3)	(4)	(5)	(6)	(7)	(8)	(9)	(10)	(11)	(12)
(1) PALS_MAS	**0.754**											
(2) PALS_PER	0.004	**0.752**										
(3) PALS_PAV	0.061	0.319	**0.823**									
(4) PPASS	−0.021	−0.023	0.086	**0.701**								
(5) CVA_CTR	0.184	−0.074	0.003	0.054	**0.729**							
(6) CVA_VAL	0.265	−0.013	−0.015	0.183	0.238	**0.754**						
(7) AEQ_POS	0.245	0.020	0.120	0.031	0.186	−0.004	**0.744**					
(8) AEQ_NEG	−0.005	0.157	0.191	0.013	−0.155	−0.160	−0.065	**0.711**				
(9) CVS_COL	0.102	−0.009	0.028	0.243	−0.018	0.007	−0.013	−0.037	**0.731**			
(10) CVS_PWD	0.035	0.050	0.216	−0.021	0.032	0.019	0.008	0.010	−0.051	**0.817**		
(11) SWLS	0.152	0.018	0.035	0.012	0.253	0.156	0.388	−0.349	−0.006	0.011	**0.694**	
(12) CVS_UAV	0.022	−0.045	0.040	0.008	−0.002	0.001	0.061	0.008	0.048	0.001	0.054	**0.925**

Bold values on the diagonal are the square roots of AVEs for each latent variable; the remaining values are Pearson correlation coefficients.

### 4.3 Cross-group measurement invariance testing

Before comparing the mean differences and path coefficients between the Chinese and Kazakhstani surveyed students, it is first necessary to verify the Measurement Invariance (MI) of the measurement model across the two groups. This study employed Multi-Group Confirmatory Factor Analysis (MGCFA) for testing. Given that this study comprises a total of 60 measurement items, to avoid non-convergence in factor estimation, this study adopted an Item Parceling strategy at this stage following structural parsimony principles (Little et al., 2002). The reduced integrated composite variables were imported into the multi-group model to sequentially test configural invariance, metric invariance, and scalar invariance. The fit results are shown in Table 6.

**Table 6:** Fit comparison results for measurement invariance nested models.

Invariance model	χ^2^	df	χ^2^/df	CFI	TLI	RMSEA	SRMR
Model 1: Configural invariance	213.065	38	5.61	0.937	0.915	0.053	0.042
Model 2: Metric invariance	221.185	44	5.03	0.936	0.916	0.050	0.045
Model 3: Scalar invariance	249.635	50	4.99	0.928	0.912	0.049	0.048

Configural invariance model baseline χ^2^ = 213.065, df = 38; Metric invariance constrains factor loadings to be equal, χ^2^ increment is 8.12; Scalar invariance further constrains item intercepts to be equal, χ^2^ increment is 28.45. ΔCFI ≤ 0.010, ΔRMSEA ≤ 0.015 are the criteria for establishing invariance (Cheung and Rensvold, 2002).

Configural Invariance (Model 1): The configural invariance model allows the factor loadings and item intercepts to be freely estimated in both groups, only constraining the factor structure to be identical across groups. The overall model fit indices reached an acceptable level (CFI = 0.937, RMSEA = 0.053, Standardized Root Mean Square Residual (SRMR) = 0.042), indicating that the Chinese and Kazakhstani surveyed students share the same factor structure across all measurement constructs.

Metric Invariance (Model 2): Building on configural invariance, factor loadings were further constrained to be equal across the two groups. Compared to Model 1, ΔCFI = 0.001, which is below the 0.010 judgment threshold, and RMSEA also improved, indicating that the factor loadings of all items possess equivalence across the Chinese and Kazakhstani groups, supporting the comparison of path coefficients between the two groups.

Scalar Invariance (Model 3): Building on metric invariance, item intercepts were further constrained to be equal across the two groups. Compared to Model 2, ΔCFI = 0.008, ΔRMSEA = −0.001, both meeting the criteria, indicating that item intercepts are equivalent across the two groups. The establishment of scalar invariance provides the measurement foundation for subsequent latent mean comparisons.

During the progressive constraints of the measurement model, the trajectories of changes in major fit indices and non-compatibility indices are shown in Figure 2.

**Figure 2:**
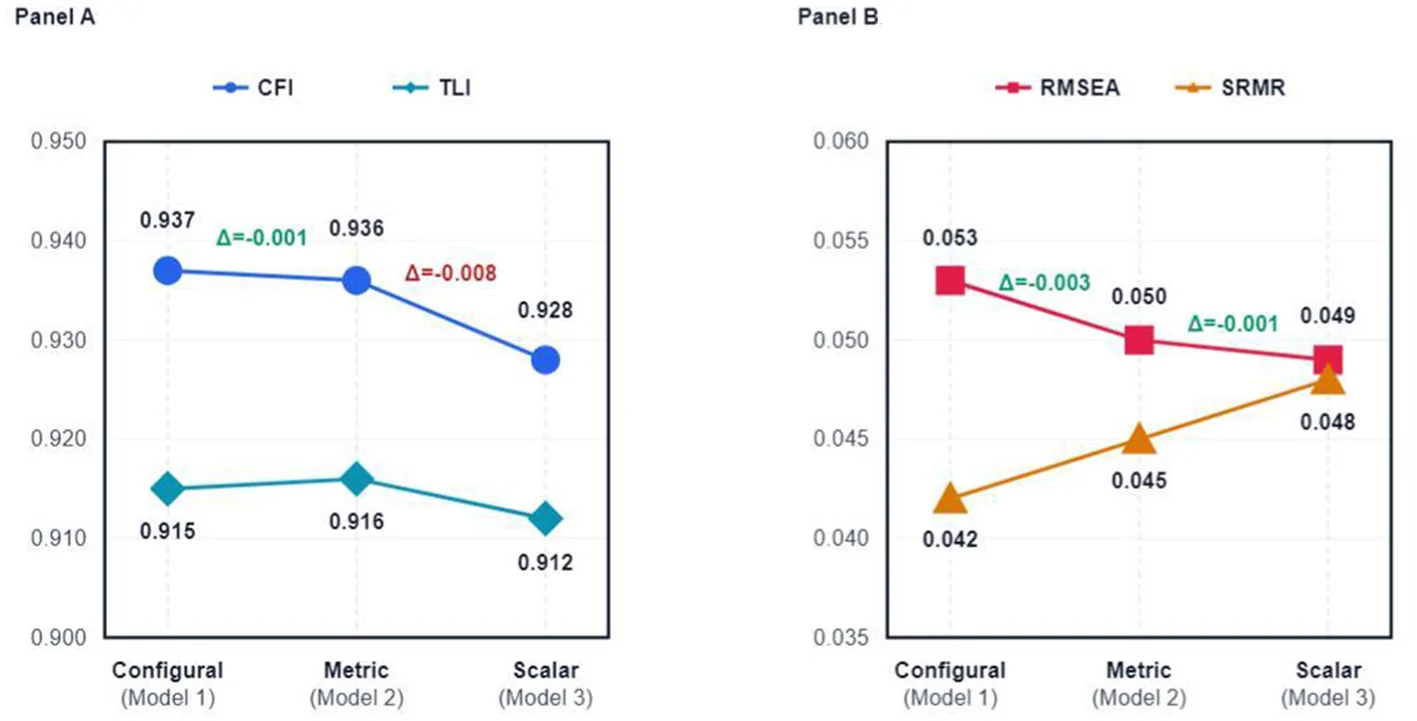
Line chart of changes in fit indices (CFI/TLI/RMSEA/SRMR) for measurement invariance testing of Chinese and Kazakhstani university students. **(A)** Goodness-of-Fit Indices (CFI & TLI). **(B)** Incompatibility Indices (RMSEA & SRMR).

In summary, the measurement model achieved strong measurement invariance across the Chinese and Kazakhstani groups, satisfying the statistical prerequisites for cross-group mean comparisons and path coefficient comparisons.

### 4.4 Descriptive statistics, normality testing, and cross-cultural variable comparisons

#### 4.4.1 Normality testing

This study examined the distributional characteristics of all composite mean variables. The absolute values of skewness for all composite variables were below 0.85, and kurtosis absolute values were below 1.10, meeting the conventional judgment criteria for univariate normal distribution, establishing the basic assumption for subsequent parametric statistical inference.

##### 4.4.1.1 Mean difference comparisons between Chinese and Kazakhstani surveyed students

The study conducted independent samples *t*-tests, using Cohen's *d* as the effect size estimation indicator. The results of the difference tests on learning ecology perceptions and academic emotion dimensions between the two groups are summarized in Table 7.

**Table 7:** Mean difference comparisons of learning ecology and academic emotion variables between Chinese and Kazakhstani surveyed university students.

Variable/dimension	China mean (*n* = 865)	China *SD*	Kazakhstan mean (*n* = 780)	Kazakhstan *SD*	*t*	*p*	Cohen's *d*	Direction of difference
PALS_MAS (mastery climate)	3.79	0.68	3.49	0.72	8.55	< 0.001^***^	0.42	China group mean higher
PALS_PER (performance-approach climate)	3.54	0.68	3.18	0.72	10.44	< 0.001^***^	0.52	China group mean higher
PALS_PAV (performance-avoidance climate)	3.32	0.82	3.04	0.76	7.21	< 0.001^***^	0.35	China group mean higher
PPASS (parental expectations and support)	3.96	0.66	3.35	0.68	18.59	< 0.001^***^	0.92	China group mean higher
CVA_CTR (perceived control)	3.27	0.69	3.10	0.68	5.03	< 0.001^***^	0.25	China group mean higher
CVA_VAL (task value)	3.73	0.69	3.54	0.71	5.54	< 0.001^***^	0.27	China group mean higher
AEQ_ENJ (enjoyment)	3.24	0.80	3.46	0.79	−5.60	< 0.001^***^	−0.28	Kazakhstan group mean higher
AEQ_HOP (hope)	3.37	0.77	3.29	0.78	2.10	0.036^*^	0.10	China group mean higher
AEQ_PRI (pride)	3.42	0.77	3.80	0.75	−10.05	< 0.001^***^	−0.50	Kazakhstan group mean higher
AEQ_POS (positive academic emotions)	3.35	0.59	3.52	0.60	−5.853	< 0.001^***^	−0.29	Kazakhstan group mean higher
AEQ_NEG (negative academic emotions)	2.87	0.49	2.74	0.47	5.543	< 0.001^***^	0.27	China group mean higher
AEQ_ANX (anxiety)	3.08	0.73	2.78	0.73	8.49	< 0.001^***^	0.42	China group mean higher
AEQ_SHA (shame)	3.05	0.79	2.56	0.78	12.66	< 0.001^***^	0.62	China group mean higher
AEQ_BOR (boredom)	2.74	0.79	3.16	0.79	−10.75	< 0.001^***^	−0.53	Kazakhstan group mean higher
AEQ_ANG (anger)	2.62	0.79	2.48	0.78	3.75	< 0.001^***^	0.19	China group mean higher
CVS_COL (collectivism)	3.54	0.64	3.21	0.69	9.95	< 0.001^***^	0.49	China group mean higher
CVS_PWD (power distance)	3.24	0.79	3.43	0.76	−4.81	< 0.001^***^	−0.24	Kazakhstan group mean higher
CVS_UAV (uncertainty avoidance)	3.67	0.84	3.55	0.88	2.62	0.009^**^	0.13	China group mean higher
SWLS (life satisfaction)	4.02	0.70	4.55	0.67	−15.72	< 0.001^***^	−0.77	Kazakhstan group mean higher

^*^p < 0.05.

^**^p < 0.01.

^***^p < 0.001.

To visually present the magnitude and direction of cross-cultural differences across core variables, the study further plotted a forest plot of standardized effect sizes (Cohen's *d*) and their 95% confidence intervals, as shown in Figure 3.

**Figure 3:**
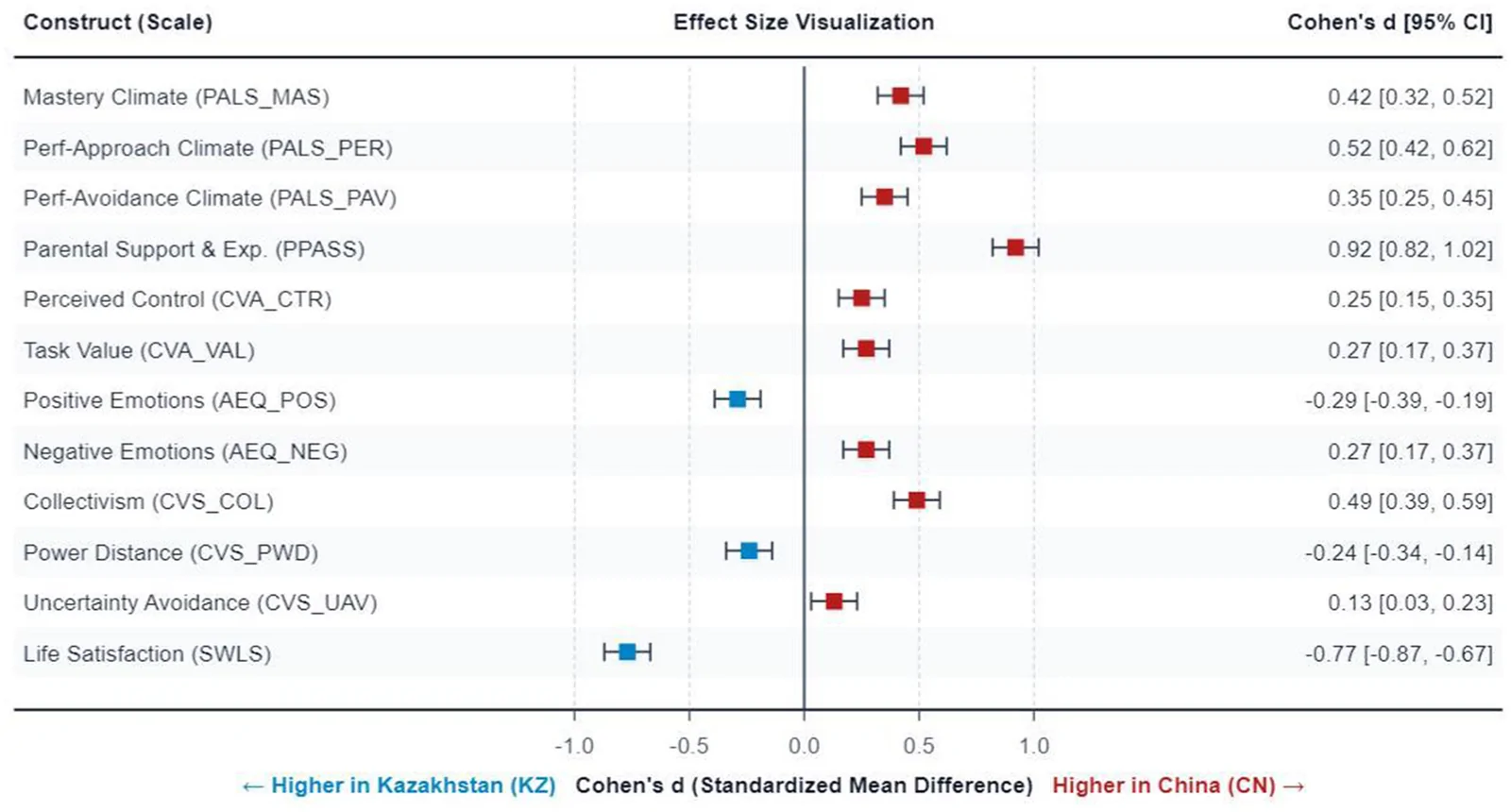
Forest plot of effect sizes for mean differences of core variables between Chinese and Kazakhstani university students.

The zero-order correlation matrices of the two sample groups provided preliminary covariation evidence for the series of research hypotheses. The significant positive association between mastery climate and academic enjoyment, as well as the positive covariation between performance-avoidance climate and anxiety and shame, were evident among students from both countries, confirming the relationships described in Hypotheses H1a and H1b. Regarding the performance-approach climate, its overall positive association with negative academic emotions remained consistent across students from both countries; however, on the perceived control dimension, the association patterns diverged between the two groups: there was no significant correlation in the Chinese sample, while a significant negative covariation emerged in the Kazakhstani sample, reflecting complex covariation characteristics accompanied by specific contexts (corresponding to Hypothesis H1c). For the family ecology, the overall index of parental expectations and support had no direct statistical association with general negative academic emotions in either country, but was positively associated with the anxiety sub-dimension; whether this family involvement environment has an indirect link with lower negative academic emotions through the mediating pathway of task value (corresponding to Hypothesis H2b) relied on verification through subsequent structural equation modeling.

### 4.5 Multi-group path analysis model (MG-SEM)

To systematically examine the relational structure wherein classroom climate and parental expectations (external environmental variables), via cognitive appraisals (perceived control and task value) and two types of achievement emotions (positive and negative), affect life satisfaction within the structural equation framework, this study fitted a multi-group path analysis model containing complete control-value predictive pathways. The overall fit indices of the model met accepted criteria (CFI = 0.920, Tucker-Lewis Index (TLI) = 0.916, RMSEA = 0.046, SRMR = 0.045). The final estimated model structure is shown in Figure 4; to highlight key points, only main paths reaching statistical significance are marked in the figure, and cross-cultural differences are differentiated by colors. Full details of all precise path parameters and inter-group comparisons of the complete model can be seen in Table 8.

**Figure 4:**
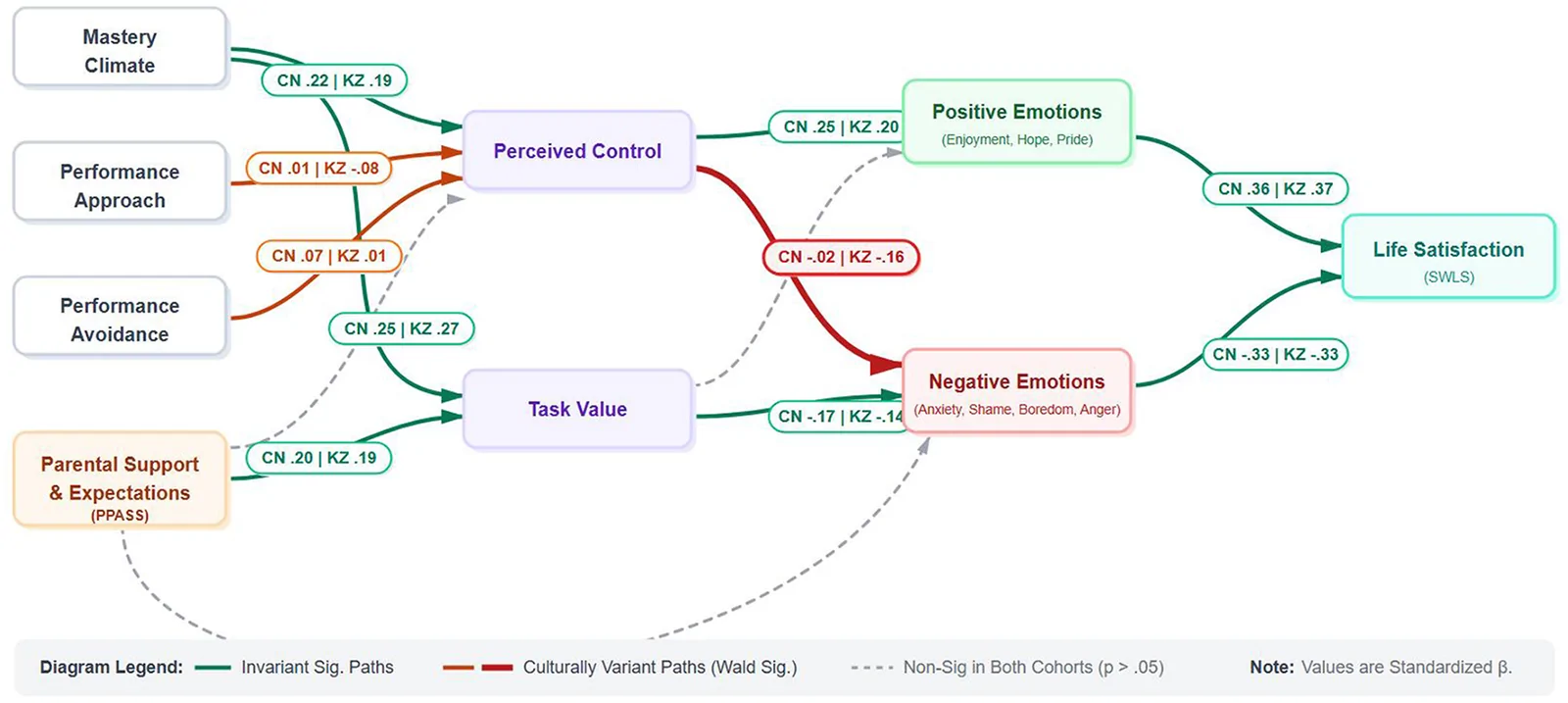
Standardized coefficient model diagram of multi-group path analysis for Chinese and Kazakhstani university students.

**Table 8:** Comparison of core path parameters of the multi-group model for the surveyed samples in China and Kazakhstan.

Path	China β	*p*	Kazakhstan β	*p*	Wald χ^2^	CRD
CVA_CTR ← PALS_MAS	0.224	< 0.001^***^	0.185	< 0.001^***^	0.647	0.804
CVA_CTR ← PALS_PER	0.008	0.815	−0.077	0.038^*^	2.803	1.674
CVA_CTR ← PALS_PAV	0.070	0.046^*^	0.011	0.768	1.326	1.152
CVA_CTR ← PPASS	0.015	0.660	0.056	0.116	0.716	−0.846
CVA_VAL ← PALS_MAS	0.245	< 0.001^***^	0.269	< 0.001^***^	0.255	−0.505
CVA_VAL ← PPASS	0.195	< 0.001^***^	0.189	< 0.001^***^	0.017	0.131
AEQ_POS ← CVA_CTR	0.250	< 0.001^***^	0.198	< 0.001^***^	1.075	1.037
AEQ_POS ← CVA_VAL	−0.044	0.197	−0.051	0.158	0.022	0.149
AEQ_NEG ← CVA_CTR	−0.015	0.659	−0.156	< 0.001^***^	8.204^**^	2.864^**^
AEQ_NEG ← CVA_VAL	−0.166	< 0.001^***^	−0.139	< 0.001^***^	0.556	−0.746
AEQ_NEG ← PPASS	0.034	0.320	0.021	0.552	0.067	0.259
SWLS ← AEQ_POS	0.358	< 0.001^***^	0.367	< 0.001^***^	0.045	−0.212
SWLS ← AEQ_NEG	−0.328	< 0.001^***^	−0.325	< 0.001^***^	0.004	−0.066

Path parameters are all standardized regression coefficients (β). CRD is the critical ratio difference value estimated based on unstandardized coefficients and standard errors; |CRD| > 1.96 suggests cross-group path differences reach the *p* < 0.05 level. Wald χ^2^ (df = 1) > 3.84 also indicates a significant difference in path coefficients across groups.

^*^p < 0.05.

^**^p < 0.01.

^***^p < 0.001.

#### 4.5.1 Association between classroom climate and cognitive appraisals

As illustrated in the left panel of Figure 4, mastery climate (PALS_MAS) exhibited a robust positive association crossed culturally consistent with both perceived control (CVA_CTR) and task value (CVA_VAL) in both samples (green solid lines). However, the path between performance-approach climate (PALS_PER) and perceived control displayed completely different characteristics in the two countries (orange solid line): there was no significant statistical correlation in the Chinese sample, whereas a significant negative covariation relationship emerged in the Kazakhstani sample. This finding demonstrates the intricate situational specificity of the double-edged sword characteristic between a competitive-oriented climate and individual self-efficacy, supporting Hypothesis H1c.

Association between Parental Expectations and Support and Cognitive Appraisals At the family ecology level, parental expectations and support (PPASS) and task value cognition demonstrated a robust positive covariation mechanism in both samples. However, the gray dashed line in Figure 4 shows no significant direct linkage between PPASS and perceived control in either sample group, thereby failing to support the expectation in Hypothesis H2a regarding a negative association between the two. Moreover, under conditions controlling for other variables, the direct association path between PPASS and negative academic emotions (bottom long dashed line) was also non-significant in both countries, confirming that family involvement primarily relies on the mediating pathway of “enhancing value appraisals” to indirectly associate with negative academic emotions, thus supporting the indirect association logic argued in Hypothesis H2b.

Association of Emotions on Life Satisfaction In the transmission network from cognition to emotions, a stable positive cross-group equivalent association (green solid line) emerged between perceived control and positive emotions, whereas the direct association path of task value on positive emotions did not reach a significant level in either country (gray dashed line). Looking at the spillover phase from academic emotions to overall life adaptation, positive emotions, negative emotions, and life satisfaction exhibited significant positive and negative covariation characteristics, respectively, and these two core pathways (the final green solid lines on the right) demonstrated high cross-cultural consistency. This clear ecological indirect statistical association pathways fully supports Hypothesis H4.

Cross-Group Differences in the Association between Perceived Control and Negative Emotions The notably bolded red path in Figure 4 (Perceived Control → Negative Emotions) reveals the most central cross-cultural heterogeneous characteristic in this study. The significant negative covariation association between perceived control and negative emotions existed only in the Kazakhstani sample, whereas in the Chinese sample, the covariation relationship between the two was extremely weak and non-significant. The inter-group critical ratio difference test (CRD = 2.864 > 1.96) statistically confirmed that this path possessed substantial non-equivalence, profoundly substantiating the complex cultural variability of the “control-negative emotion” covariation pattern across the two types of educational ecologies.

### 4.6 Moderation and mediation effect analysis

#### 4.6.1 Bootstrap confidence interval estimation of mediation effects

To test the mediating roles of perceived control (CVA_CTR) and task value (CVA_VAL) between classroom climate and academic emotions, this study employed a bias-corrected Bootstrap method (5,000 resamples) to estimate indirect effects.

Regarding the mediation path “Mastery-oriented (PALS_MAS) → Perceived Control (CVA_CTR) → Positive Achievement Emotions (AEQ_POS),” the standardized indirect statistical association pathways for the Chinese university student group was 0.0562, with a 95% confidence interval of [0.0333, 0.0837]; the indirect statistical association pathways value for the Kazakhstani group was 0.0367, with a 95% CI of [0.0193, 0.0585]. The confidence intervals for both groups did not contain 0, indicating that the mediating association of perceived control between mastery climate and positive emotions was statistically significant in both country samples.

Further testing the mediating protective pathway for negative academic emotions: in the path “Mastery Climate (PALS_MAS) → Task Value (CVA_VAL) → Negative Achievement Emotions (AEQ_NEG),” the standardized indirect statistical association pathways for the Chinese sample was −0.0417, with a 95% confidence interval of [−0.0637, −0.0223]; the standardized indirect statistical association pathways for the Kazakhstani sample was −0.037, with a 95% confidence interval of [−0.0721, −0.0277].

In the path “Parental Expectations and Support (PPASS) → Task Value (CVA_VAL) → Negative Achievement Emotions (AEQ_NEG),” the standardized indirect statistical association pathways for the Chinese sample was −0.0331, with a 95% confidence interval of [−0.0509, −0.0179]; the standardized indirect statistical association pathways for the Kazakhstani sample was −0.026, with a 95% confidence interval of [−0.0542, −0.0178]. The aforementioned confidence intervals did not contain 0, indicating that both classroom mastery climate and parental expectations and support can link to lower levels of negative academic emotions by enhancing individuals' perceived task judgments and value identification regarding academics, and this indirect relational mechanism was significant in both country samples, partially supporting Hypothesis H3 with data. Notably, although the zero-order correlation between family expectations and support (PPASS) and negative academic emotions (AEQ_NEG) was close to zero, the mediation effect here was significant. Under mediation testing criteria, this phenomenon manifests as a typical “Indirect-only mediation.” This suggests that the configuration of parental expectations and support does not possess direct covariation attributes with negative academic emotions, but can only exert a weakly associated protective role indirectly by stimulating an individual's single transmission pathway of task judgment and value identification toward academics.

Furthermore, because the direct path from task value to positive academic emotions was not significant in either sample, the indirect pathway “Classroom Climate/Parental Expectations and Support → Task Value → Positive Academic Emotions” did not obtain statistically significant support, indicating an asymmetry in the cognitive appraisal mediation model across positive and negative emotional pathways. The Bootstrap estimates and 95% confidence interval distributions of the three core indirect pathways across the Chinese and Kazakhstani surveyed samples are shown in Figure 5.

**Figure 5:**
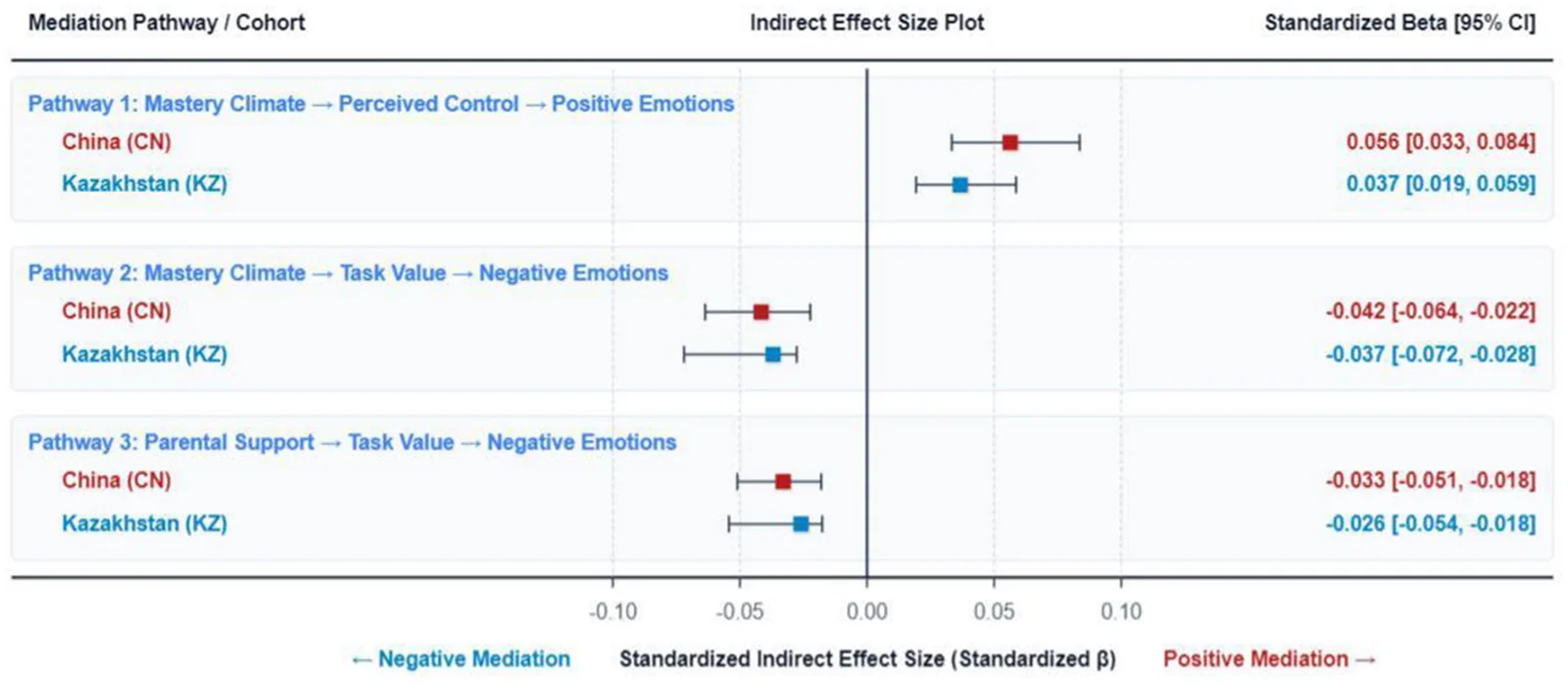
Forest plot of bootstrap estimates for indirect effects of core mediation pathways among Chinese and Kazakhstani surveyed students.

#### 4.6.2 Analysis of the moderating effect of collectivism and power distance

To test whether collectivist values (CVS_COL) and power distance (CVS_PWD) play a moderating role between parental expectations and academic support (PPASS) and negative academic emotions (AEQ_NEG), this study used data from the Chinese surveyed group and included the centered PPASS, cultural value variables, and their interaction terms into a hierarchical regression. The analyses were modeled for the Chinese and Kazakhstani cohorts separately. The results are shown in Table 9.

**Table 9:** Hierarchical regression analysis of the moderating role of collectivism on the relationship between parental expectations and negative academic emotions (cross-cultural comparison).

Predictor variables	*B*	*SE*	β	*t*	*p*
Chinese sample					
Constant	2.885	0.017	—	167.88	< 0.001^***^
PPASS	0.042	0.026	0.057	1.60	0.109
CVS_COL	−0.023	0.027	−0.030	−0.84	0.401
PPASS × CVS_COL	0.045	0.041	0.038	1.10	0.272
Kazakhstani sample					
Constant	3.132	0.015	—	210.54	< 0.001^***^
PPASS	−0.040	0.019	−0.078	−2.17	0.030^*^
CVS_COL	0.018	0.018	0.034	0.96	0.338
PPASS × CVS_COL	0.010	0.023	0.015	0.41	0.681

Before including interaction terms, all predictor variables and interaction term constructions underwent standard reverse-scoring conversions and mean-centering. The dependent variable is the composite mean of negative academic emotions (AEQ_NEG).

^*^p < 0.05.

^***^p < 0.001.

The results showed that after correct psychometric conversion processing of the CVS_COL reverse items (CVS_COL_04_R), the interaction term coefficient of PPASS and CVS_COL did not reach statistical significance (*B* = 0.045, *t* = 1.10, *p* = 0.272); concurrently, the test for the interaction term of power distance (CVS_PWD) was similarly non-significant (*p* = 0.238).

Crucially, the structural pathways tested in the independent Kazakhstani cohort yielded consistent non-significant results. As shown in the lower section of Table 9, neither the collectivism interaction term (*B* = 0.010, *p* = 0.681) nor the unlisted power distance interaction term (*p* = 0.685) demonstrated significant moderating associations.

This cross-cultural alignment suggests that collective values did not significantly moderate the statistical association between parental academic expectations and negative academic emotions in the surveyed samples, thus yielding no empirical support for Hypothesis H5. We initially hypothesized that Confucian high power distance or nomadic egalitarian collectivism might contextualize and soften the immediate emotional weight of familial academic pressure. However, the observed data structure reflects a much starker reality: in the contemporary climate of higher education competition, high parental expectations structurally correspond to an immediate emotional burden. It appears that deeply rooted cultural values—whether traditional filial piety or community solidarity—may no longer effectively shield or buffer the immediate anxiety structurally tied to parental academic demands.

## 5 Discussion

### 5.1 Association between classroom goal climate and cognitive appraisals

Path model results indicated a stable positive covariation relationship between classroom mastery climate and perceived control as well as task value in both samples, with no statistically significant cross-group differences between China and Kazakhstan regarding this association. This result is consistent with the core assumption of the control-value theory (Pekrun, 2006): a classroom goal structure emphasizing self-growth and knowledge internalization is associated with an individual's higher mastery expectations and positive emotional experiences. However, the absolute values of the path coefficients were in the small to medium effect size range, and the proportion of variance in individuals' cognitive appraisals explained by classroom climate variables was limited, a phenomenon present in both national samples.

One possible explanation relates to the applicable context of the PALS scale. This tool was developed by Midgley et al. (2000) with fixed class systems in basic education stages as the reference background, and its items presuppose students' continuous perception of a single, stable classroom climate. Top-tier Chinese universities and Kazakhstani universities undergoing Bologna Process reforms have generally implemented course selection and moving-class systems, meaning students concurrently attend multiple classes with varying goal orientations. In this context, what PALS items capture may be heterogeneous averages derived from different classrooms rather than the true state of a single stable unit, thereby statistically diluting the association strength between classroom climate variables and cognitive appraisals. Future research could potentially improve the ecological validity of this tool in higher education contexts by anchoring PALS measurements to specific courses or a single teacher.

The divergent associations between performance-approach climate (PALS_PER) and perceived control exhibited in the two samples deeply reflect the situational variability of the double-edged sword characteristic hypothesized in H1c. In the Chinese sample, the statistical decoupling between performance-approach climate and autonomous control may stem from the highly selective competitive environment having become a normative background in the higher education ecology of the Confucian cultural sphere; consequently, Chinese university students have established a contextual buffering cognition, resulting in a low degree of association between intra-class ranking competition and internal locus of control. Conversely, in the Kazakhstani sample, experiencing educational transitions and variable academic rules, high-level performance demands intertwined with rule uncertainty are highly prone to manifest a negative association with individual self-efficacy reduction. By unpacking this phenomenon through the integrated lens of EST and CVT, we observe how macrosystem instability (educational transition) infiltrates the school microsystem, subsequently altering the functional direction of cognitive appraisals. This suggests that in higher education contexts, the association mechanism between performance-approach climate and psychological adaptation appraisals varies contextually based on cultural and institutional backgrounds, and should not be simply reduced to a single adaptive or maladaptive variable. Hawrot's (2024) longitudinal design-based research also indicates that long-term interactions of control and value appraisals shape the evolutionary path of emotions, supporting the necessity of simultaneously investigating multiple cognitive appraisal pathways.

### 5.2 Association between family academic expectations and cognitive appraisals

The PPASS scale used in this study focused on parents' academic expectations, daily monitoring, and proactive assistance provided when facing difficulties. The initial path structure and cross-cultural baseline comparison parameters were systematically calibrated. The underlying rationale is elucidated as follows:

Path analysis results showed that this composite family input could reflect academic value feedback that “learning is related to the future,” demonstrating a significant, cross-culturally consistent positive association on the task value channel. However, on the perceived control channel, the statistical association between the two variable groups was non-significant, leading to the failure of data to support Hypothesis H2a (parental expectations and support are significantly negatively associated with perceived control). This decoupling phenomenon stems from the interdependent interaction model under Asian and Central Asian cultural contexts: high-intensity performance monitoring and expectations are internalized by students as closely homologous to resource support within a “family interdependent system” (the high internal consistency reliability of the scale directly confirms the inseparable correlational attribute of the two). Therefore, PPASS did not generate offsetting positive and negative validities internally; rather, this composite support system itself, possessing strong family interdependence, though cultivating a “sense of task value” to repay the family, logically and objectively failed to transform into the young individual's “independent personal autonomous control” stripped from family parasitism.

From a cross-cultural comparison, the overall level of perceived parental expectations and academic support (PPASS) among the Chinese group students was significantly higher than that of the Kazakhstani group, with the largest effect size among all inter-group mean differences in this study (*d* = 0.92). Despite the significant gap in intensity, the transmission network between this system and individual cognitive appraisals displayed a consistent structural pattern across the two countries, confirming the high cross-sample stability of this family microsystem association model (Zeng et al., 2024).

Additionally, regarding the relationship between parental academic expectations/support and negative emotions, Hypothesis H2b was not supported along the direct effect path to the overall index of negative emotions. Based on the previously mentioned cultural interdependent attributes, monitoring (controlling input) and support (resource input) are already integrated into the individual's cognition, existing as a composite background without directly accompanying unidirectional emotional collapse. This suggests that the logical pivot of university family intervention needs to shift: there is no need to excessively force a mechanical separation between parental monitoring and support, but rather, from an ecological perspective, focus on buffering the pressure radiating from the family microsystem. Assisting young students in establishing an endogenous efficacy evaluation baseline independent of family expectations.

### 5.3 Moderating effect of collectivist values

To structurally test the boundary conditions postulated by macro-cultural frameworks, our revised hierarchical moderation analysis examined whether collectivist values (CVS_COL) and power distance (CVS_PWD) buffered the association between family academic expectations (PPASS) and negative emotions. The empirical results yielded a profound sociological alignment: these cultural values displayed no statistically significant moderating role in either the Chinese or the Kazakhstani cohorts. This finding suggests that, in the higher education ecology, traditional macro-cultural values have evolved from “sensitive situational moderating variables” to broad and stable “background variables” among university student populations.

The potential logic causing this mechanism is primarily related to the unique psychological developmental stage of the university student demographic and the shared structural severity of modern academic ecosystems. University students transitioning into “early adulthood” exhibit stronger needs for individuation and psychological independence. Regardless of whether they were socialized in a Confucian hierarchy (China) or a nomadic egalitarian tradition (Kazakhstan), their immediate negative academic emotional states (such as anxiety and shame) are structurally tied to systemic academic competition and self-efficacy evaluations. Traditional cultural beliefs have settled into the “normalized background” of students' emotional processing, making it difficult to induce differential risk modulation effects internally. In contemporary highly selective higher education ecologies across both regions, the stress-inducing nature of parental expectations constitutes a direct and overwhelming structural burden. Distal macro-cultural beliefs appear to lack sufficient resilience to shield or buffer this proximal, overwhelming emotional load.

### 5.4 Cross-cultural applicability of control-value theory in sino-kazakh educational ecologies

As the core theoretical framework of this study, CVT demonstrated a substantial degree of cross-cultural robustness across the Chinese and Kazakhstani university student samples: mastery climate showed a positive indirect association via perceived control with positive emotions; positive emotions correlated positively with life satisfaction; and negative emotions correlated negatively with life satisfaction. These core associations obtained statistical support in both datasets, with cross-group differences in path coefficients being non-significant, indicating that CVT possesses certain theoretical applicability in higher educational contexts of differing cultural backgrounds (Pekrun et al., 2007). It is noteworthy that the path from task value to positive emotions was not significant in either group, showing a partial deviation from the ‘dual parallel pathways' assumption of classic CVT. This might be related to the phased characteristics of higher education: university students' academic value evaluations often carry strong long-term instrumentality (such as Grade Point Average (GPA) or employment intentions). Applying EST and CVT to unpack this phenomenon indicates that while task value connects cognitively to broad macro-system trajectories (e.g., long-term career paths), it fails to structurally associate with immediate, situated positive affects (like ‘enjoyment') in the micro-classroom setting, which heavily rely on proximal, immediate sense of control. This underscores a critical boundary condition of the CVT when applied to older students or higher education—instrumental values do not necessarily translate to immediate experiential states. This finding suggests that the application of CVT in higher education contexts should consider the asymmetry between different cognitive appraisals and emotional connections. Furthermore, this demonstrates an ecological spillover effect between emotions and life satisfaction, consistent with Feraco et al.'s (2023) model, namely that within an individual's developmental ecosystem, the emotional experiences of the academic microsystem will deeply spill over into the individual's evaluation of global life satisfaction.

However, there are boundaries to the cross-cultural applicability of CVT. This study identified a significant cross-group difference in the association between perceived control and negative emotions: this path was significantly established in the Kazakhstani sample but approached zero in the Chinese sample. By unpacking this mechanism through the intertwined theories, we can articulate why this structural divergence occurs. During the institutional uncertainty of Kazakhstan's higher educational modernization transition, students' lack of control is more likely to be attributed to the unpredictability of the external environment, thereby associating with stronger experiences of anxiety and helplessness. In contrast, Chinese university students have long been situated in a competitive academic environment; the cultural tendency for “effort attribution” (Dweck, 1999) may lead them to interpret low feelings of control as “needing to work harder,” Consequently, the macro-cultural endorsement of “effort attribution” in China acts as a cognitive buffer within CVT's appraisal mechanism: it drives individuals to internalize control loss as an actionable, personal striving deficiency rather than a definite institutional threat, structurally mitigating the direct statistical association with immediate emotional collapse.

This finding suggests that the fundamental assumption in CVT—that emotions are primarily shaped by cognitive appraisals—may interact with individual-level cultural coping styles, thereby altering the statistical significance landscape on specific pathways. Future cross-cultural studies might attempt to systematically incorporate cognitive reappraisal tendencies as moderating variables within the CVT framework to test the application boundaries of this theory across different cultural backgrounds.

## 6 Conclusions and implications

### 6.1 Main conclusions of the study

Using the Control-Value Theory and Ecological Systems Theory as an integrated framework, and based on cross-cultural survey data from a total of 1,645 enrolled university students in China and Kazakhstan, this study systematically examined the structural associations among classroom goal climate, parental expectations and academic support, cognitive appraisals, academic emotions, and life satisfaction, and verified the cross-group measurement invariance of the instruments between the two national samples.

The study reached the following core conclusions:

First, the association between classroom mastery climate and positive academic emotions is primarily mediated by the perceived control channel. Mastery climate establishes a stable positive association with university students' positive academic emotions via perceived control, the core cognitive appraisal channel, whereas the mediating role of perceived task value in this positive emotional pathway is not significant. Concurrently, mastery climate can also demonstrate significant negative association with individuals' lower negative academic emotions via the task value pathway, serving as an important factor associated with low negative academic maladjustment. The association in this effective control channel showed no significant path differences between the Chinese and Kazakhstani groups, indicating cross-cultural consistency in how classroom designs emphasizing self-growth and knowledge internalization correlate with high academic mastery expectations and positive emotions in university students.

Second, a systematic decoupling exists between family expectation support and perceived control, strongly related to instrumental/utilitarian value cognition. Under the pan-Asian cultural ecology, parents' high expectation monitoring and resource supply exhibit highly interdependent cognitive binding. This support system (PPASS) is significantly positively associated with an individual's instrumental evaluation of academic importance, forming a protective pathway via the value feeling channel that indirectly links to lower-level negative emotions. However, given the strong “family interdependent” attribute of this support system, it cannot directly translate into university students' “personal autonomous control.” It is not that parental expectations do not generate control pressure; rather, what is typically viewed as oppressive ‘monitoring' in Western contexts is inextricably wrapped in ‘resource support' in Eastern and Central Asian contexts. Consequently, its undermining effect on an individual's autonomous control is offset or masked. The discovery of this pathway reveals the complexity and boundaries of family academic involvement in cross-cultural contexts.

Third, academic emotions act as a crucial bridge in the relational loop of classroom/family ecological perceptions and life satisfaction. The positive association between positive emotions and life satisfaction, and the negative association between negative emotions and life satisfaction, exhibited highly similar associational strengths in both countries, substantiating the critical role of the spillover effect of academic emotions in the psychological adaptation of young people during the university stage.

Fourth, the structural homogenization of macro-cultural buffering. Neither family expectation-oriented collectivism nor power distance demonstrated significant moderating and buffering capacities in the association between parental expectations and academic support (PPASS) and negative emotions across both cohorts. This characteristic negates the moderation hypothesis and instead suggests a cross-cultural consistency and stability of the associational paths between family microsystems and emotional adaptation across individuals with different cultural orientations, indicating that macro-values do not serve as the main adjusting valve for the immediate emotional relief systems of current university students in China and Kazakhstan in the face of systemic academic competition.

### 6.2 Implications for higher education intervention and mental health management

Classroom level: Construct growth-oriented goal structures. The cross-culturally stable association of mastery climate with cognitive appraisals and positive emotions suggests that university teacher training systems should incorporate contents related to goal climates, guiding teachers to reduce rank-competition orientations in course design, feedback language, and evaluation criteria, shifting focus to students' cognitive developmental processes. In higher education environments where elective systems prevail, the micro-goal climate within a single course possesses independent psychological significance; one cannot expect students to spontaneously form mastery-oriented cognition merely by integrating impressions across multiple classes.

Family level: Promote a shift from monitoring-based involvement to specific support-based involvement. The data in this study show that expectations mixed with monitoring and expressions of disappointment cannot translate into students' perceptions of autonomous control. University family education guidance should impart non-intrusive support concepts, guiding parents to provide more specific support based on overcoming difficulties, and reduce the use of disappointment and high-pressure monitoring as means of promoting study, so as to avoid inducing excessive anxiety and shame burdens while maintaining high task value.

Mental health services level: Focus on the spillover associations of negative emotions on overall wellbeing. The negative association path of negative emotions on life satisfaction proved robust in both country samples, suggesting that university psychological counseling and health education should focus on identifying core anxiety and feelings of shame. Institutions should formulate coping plans addressing Chinese university students' bruised high self-esteem (shame) and Kazakhstani university students' relatively higher feelings of boredom. Moreover, since cultural values (such as collectivism and power distance) were not found to exhibit noticeable psychological buffering or resistance defense functions here, psychological intervention systems in both China and Kazakhstan should not over-rely on generalized cultural resilience. Instead, university counselors and families must structurally shift toward providing direct emotional coping mechanisms and autonomy-supportive parental involvement to help individuals detach from family performance bindings to achieve healthy adaptive desensitization.

### 6.3 Limitations of the study

First, limitations of causal inference due to the cross-sectional design. All data in this study were collected at a single time point; statistical associations between variables can only support statements of “systematic association” and cannot rule out reverse causality or alternative explanations from third variables. For instance, whether students with higher life satisfaction inherently tend to report more positive emotions, and whether a bidirectional association exists between perceived classroom mastery climate and perceived control, cannot be distinguished within a cross-sectional design framework. Future research should consider adopting longitudinal or Experience Sampling Method (ESM) designs to track dynamic changes in cognitive appraisals and emotional states over a semester, providing stricter temporal sequence evidence for the causal chain of CVT. Future research should utilize longitudinal cross-lagged panel models or Experience Sampling Method (ESM) designs to explicitly establish temporal causality and track dynamic intra-individual changes in cognitive appraisals and emotional states over time.

Second, the risk of semantic equivalence during translation. Although this study followed standard translation-back-translation procedures, emotional and attitude items might still experience semantic drift during cross-lingual conversion. Chinese, Russian, and Kazakh differ in the granularity and cultural connotations of emotional vocabulary, potentially leading identical scale items to activate incompletely equivalent psychological content across different language groups, introducing cross-lingual measurement errors at the micro-level that are difficult to fully quantify. To address this, subsequent researchers are encouraged to apply advanced psychometric techniques, such as Differential Item Functioning (DIF) via Item Response Theory, to meticulously quantify and isolate these subtle cross-lingual noises.

Third, the limitations of geographical representation of the samples. The Chinese sample in this study mainly came from the Shanghai area, and the Kazakhstani sample primarily from Almaty and its surroundings. Both are core urban areas in their respective countries, possessing certain specificity in higher educational resources, family economic backgrounds, and cultural diversity. Generalizing the research conclusions to university student populations in rural and other regions of China and Kazakhstan requires caution, especially concerning variables sensitive to geopolitical cultural gradients, such as parental expectations and academic support and the moderating effect of collectivism. Therefore, future studies should aim for multi-tier clustered probability sampling across diverse regional and socioeconomic strata to substantially enhance ecological generalizability.

Fourth, unmeasured confounding variables. The path model in this study did not include variables such as family socioeconomic status (SES), nature of the major, grade, stage of study, and prior academic achievement levels. These variables might co-vary systematically with the main predictor variables, affecting the unbiasedness of the structural parameter estimates. Future research should include these background variables in the measurement system during the design phase and control for them as covariates to enhance the conclusive validity of the path model. Future research should rigorously include these structural background variables as covariates in the measurement design phase to enhance the conclusive validity of the path parameter estimates.

Fifth, the cultural generalization characteristics and construct boundaries of measurement tools. The parental expectations and academic support (PPASS) scale used in this study entered the model as a single composite indicator in the path analysis. Data confirmed that high expectation monitoring, considered “negative control” by Western scales, and proactive difficulty assistance, considered “positive construction,” exhibited highly integrated directional association in the data feedback from students in both China and Kazakhstan. Future cross-cultural research needs to develop or introduce measurement tools with higher cultural granularity to strictly differentiate between “pure autonomy support stripped of deprivation” and the “interdependent deep-monitoring support” unique to the East, aiding in a more precise elucidation of the differentiating functional mechanisms of family mesosystem elements on the evolution of youth independent efficacy. To more precisely elucidate the functional mechanisms of family mesosystems on youth efficacy, future scholars are highly encouraged to develop localized multidimensional scales. These tools should be specifically designed to capture the nuanced “family-oriented collectivism” and deeply embedded intergenerational moral ties indigenous to Eurasian cultures, rather than relying on generic work-based cultural scales.

## Data Availability

The original contributions presented in the study are included in the article/Supplementary material, further inquiries can be directed to the corresponding author.
