# Supplementary material for: Classroom and family ecology of academic achievement emotions: a cross-cultural comparison between China and Kazakhstan
Source: Front Psychol. 2026 Sep 17;17:1922663. doi: 10.3389/fpsyg.2026.1922663 (PMC13627071; doi:10.3389/fpsyg.2026.1922663)
Supplement: Supplementary file 1 [file Data_Sheet_1.zip › Supplementary_Materials_Questionnaire_and_Codebook.docx]

SECTION A: Survey Questionnaire

**Informed Consent Form**

Dear Participant,

This study aims to understand students’ academic emotional experiences across different cultural contexts. The survey takes approximately 15 minutes to complete.

Please confirm the following before proceeding:

I am 18 years of age or older.

I am currently enrolled as an undergraduate or postgraduate student.

I am currently studying in China (Shanghai) or Kazakhstan.

Important Notes:

This survey is completely anonymous.

Data will be used for academic research purposes only.

Participation is entirely voluntary and you may withdraw at any time without consequence.

Some questions relate to academic stress; please stop if you feel uncomfortable.

Clicking “Next Page” indicates your informed consent to participate.

If you do not consent, please close this page.

**Part Ⅰ: Basic Information**

Please select the option that best describes you. All information is used for statistical analysis only.

**Q1. Your current country/region of study:**

China (Shanghai)

Kazakhstan

**Q2. Your gender:**

Male

Female

Other

Prefer not to say

**Q3. Your age:**

18 - 20

21 - 23

24 - 26

27 and above

**Q4. Your current level of study:**

Undergraduate

Master’s Student

PhD Student

Other

**Q5. Highest education level of your parent(s)/guardian(s):**

Junior high school or below

Senior high school or vocational

Bachelor’s degree

Master’s degree or above

Not sure

**Part Ⅱ: Perceived Classroom Goal Structure**
*Please rate each statement based on your true experience of your classroom environment.*
1 = Strongly Disagree, 2 = Disagree, 3 = Neutral, 4 = Agree, 5 = Strongly Agree

*Mastery-Oriented Climate*

1.My teacher emphasizes the importance of learning new knowledge and skills. [ 1 ] [ 2 ] [ 3 ] [ 4 ] [ 5 ]

2.My teacher treats mistakes as part of learning and encourages us to grow from them. [ 1 ] [ 2 ] [ 3 ] [ 4 ] [ 5 ]

3.Improving one’s ability is valued more than getting high grades. [ 1 ] [ 2 ] [ 3 ] [ 4 ] [ 5 ]

4.My teacher encourages genuine understanding rather than rote memorization. [ 1 ] [ 2 ] [ 3 ] [ 4 ] [ 5 ]

*Performance-Oriented Climate*

1.Getting better grades than other students is very important in my class. [ 1 ] [ 2 ] [ 3 ] [ 4 ] [ 5 ]

2.My teacher frequently compares and ranks students’ grades. [ 1 ] [ 2 ] [ 3 ] [ 4 ] [ 5 ]

3.Students in my class care a lot about whether their grades are better than others. [ 1 ] [ 2 ] [ 3 ] [ 4 ] [ 5 ]

4.I feel I must prove that I am more capable than my classmates. [ 1 ] [ 2 ] [ 3 ] [ 4 ] [ 5 ]

*Performance-Avoidance Climate*

1.Students who perform poorly feel mocked or looked down upon in class. [ 1 ] [ 2 ] [ 3 ] [ 4 ] [ 5 ]

2.I am very worried about making mistakes in class because it makes me look incompetent. [ 1 ] [ 2 ] [ 3 ] [ 4 ] [ 5 ]

3.The most important thing here is to avoid looking bad. [ 1 ] [ 2 ] [ 3 ] [ 4 ] [ 5 ]

**Part Ⅲ: Perceived Parental Academic Support Scale (PPASS)**
*Please rate each statement based on your true perception of your parents’/guardians’ attitudes toward your academic life.*
1 = Strongly Disagree, 2 = Disagree, 3 = Neutral, 4 = Agree, 5 = Strongly Agree

1.My parents have high expectations for my academic performance. [ 1 ] [ 2 ] [ 3 ] [ 4 ] [ 5 ]

2.My parents frequently pay attention to my studies and grades. [ 1 ] [ 2 ] [ 3 ] [ 4 ] [ 5 ]

3.My parents believe that good academic performance is crucial for my future. [ 1 ] [ 2 ] [ 3 ] [ 4 ] [ 5 ]

4.My parents feel disappointed when my academic performance is unsatisfactory. [ 1 ] [ 2 ] [ 3 ] [ 4 ] [ 5 ]

5.My parents actively help me overcome difficulties in my studies. [ 1 ] [ 2 ] [ 3 ] [ 4 ] [ 5 ]

**Part Ⅳ: Achievement Emotions Questionnaire (AEQ)**
*Please rate each statement based on your genuine emotional experiences during learning and exams over the past semester.*
1 = Never, 2 = Rarely, 3 = Sometimes, 4 = Often, 5 = Always

Enjoyment

1.I enjoy learning new things. [ 1 ] [ 2 ] [ 3 ] [ 4 ] [ 5 ]

2.I often get absorbed in class and feel pleased. [ 1 ] [ 2 ] [ 3 ] [ 4 ] [ 5 ]

3.I feel satisfied and happy after completing a learning task. [ 1 ] [ 2 ] [ 3 ] [ 4 ] [ 5 ]

*Hope*

1.When facing a difficult task, I believe I can succeed. [ 1 ] [ 2 ] [ 3 ] [ 4 ] [ 5 ]

2.I feel optimistic about my academic future. [ 1 ] [ 2 ] [ 3 ] [ 4 ] [ 5 ]

3.Even after setbacks, I maintain a positive outlook on my studies. [ 1 ] [ 2 ] [ 3 ] [ 4 ] [ 5 ]

*Pride*

1.When I get good grades, I feel proud of myself. [ 1 ] [ 2 ] [ 3 ] [ 4 ] [ 5 ]

2.My academic achievements make me feel a sense of self-worth. [ 1 ] [ 2 ] [ 3 ] [ 4 ] [ 5 ]

3.I feel proud of the effort I put into my studies. [ 1 ] [ 2 ] [ 3 ] [ 4 ] [ 5 ]

*Anxiety*

1.I feel very nervous and anxious before exams. [ 1 ] [ 2 ] [ 3 ] [ 4 ] [ 5 ]

2.I feel overwhelmed when completing important academic tasks. [ 1 ] [ 2 ] [ 3 ] [ 4 ] [ 5 ]

3.I panic when I think about the possibility of failing. [ 1 ] [ 2 ] [ 3 ] [ 4 ] [ 5 ]

4.My mind goes blank during exams and I cannot concentrate. [ 1 ] [ 2 ] [ 3 ] [ 4 ] [ 5 ]

*Shame*

1.I feel very ashamed when I fail an exam. [ 1 ] [ 2 ] [ 3 ] [ 4 ] [ 5 ]

2.When my grades are poor, I feel I have let my family down. [ 1 ] [ 2 ] [ 3 ] [ 4 ] [ 5 ]

3.Performing poorly in front of classmates makes me feel very embarrassed. [ 1 ] [ 2 ] [ 3 ] [ 4 ] [ 5 ]

*Boredom*

4.I often feel bored in class and find it hard to stay interested. [ 1 ] [ 2 ] [ 3 ] [ 4 ] [ 5 ]

5.I feel that most of what I study has no meaning for my life. [ 1 ] [ 2 ] [ 3 ] [ 4 ] [ 5 ]

6.My mind wanders during study and time seems to pass very slowly. [ 1 ] [ 2 ] [ 3 ] [ 4 ] [ 5 ]

*Anger*

1.I feel dissatisfied with the current academic evaluation system. [ 1 ] [ 2 ] [ 3 ] [ 4 ] [ 5 ]

2.I feel angry when I put in effort but do not receive deserved recognition. [ 1 ] [ 2 ] [ 3 ] [ 4 ] [ 5 ]

3.I feel frustrated and resistant toward excessive assignments and exams. [ 1 ] [ 2 ] [ 3 ] [ 4 ] [ 5 ]

**Part Ⅴ: Cultural Values Scale**
*Please rate each statement based on your genuine beliefs and values.*
1 = Strongly Disagree, 2 = Disagree, 3 = Neutral, 4 = Agree, 5 = Strongly Agree

*Collectivism*

1.Bringing honor to my family is an important motivation for my academic efforts. [ 1 ] [ 2 ] [ 3 ] [ 4 ] [ 5 ]

2.My academic success belongs not only to me but also to my family. [ 1 ] [ 2 ] [ 3 ] [ 4 ] [ 5 ]

3.When necessary, personal interests should be subordinated to family and collective needs. [ 1 ] [ 2 ] [ 3 ] [ 4 ] [ 5 ]

4.I prioritize my personal development over family expectations. (Reverse-scored) [ 1 ] [ 2 ] [ 3 ] [ 4 ] [ 5 ]

*Power Distance*

1.Students should respect and follow their teachers’ decisions. [ 1 ] [ 2 ] [ 3 ] [ 4 ] [ 5 ]

2.Openly challenging a teacher’s views in class is impolite. [ 1 ] [ 2 ] [ 3 ] [ 4 ] [ 5 ]

3.There should be a certain distance and boundary between teachers and students. [ 1 ] [ 2 ] [ 3 ] [ 4 ] [ 5 ]

*Uncertainty Avoidance*

1.I prefer learning tasks and exam rules to be clearly defined. [ 1 ] [ 2 ] [ 3 ] [ 4 ] [ 5 ]

2.I feel very uneasy when facing uncertain academic prospects. [ 1 ] [ 2 ] [ 3 ] [ 4 ] [ 5 ]

**Part Ⅵ: Control-Value Appraisal Scale**
*Please rate each statement based on your subjective perception of your learning experience.*
1 = Strongly Disagree, 2 = Disagree, 3 = Neutral, 4 = Agree, 5 = Strongly Agree

*Perceived Control*

1.I believe that through effort I can control my academic outcomes. [ 1 ] [ 2 ] [ 3 ] [ 4 ] [ 5 ]

2.I feel I have sufficient control over my learning process. [ 1 ] [ 2 ] [ 3 ] [ 4 ] [ 5 ]

3.No matter how hard I try, my grades seem to be determined by external factors. (Reverse-scored) [ 1 ] [ 2 ] [ 3 ] [ 4 ] [ 5 ]

4.I can effectively manage my study time and methods. [ 1 ] [ 2 ] [ 3 ] [ 4 ] [ 5 ]

*Perceived Value*

1.I believe what I am learning is very important for my future. [ 1 ] [ 2 ] [ 3 ] [ 4 ] [ 5 ]

2.Learning feels meaningful and worth my time and effort. [ 1 ] [ 2 ] [ 3 ] [ 4 ] [ 5 ]

3.Working hard in my studies will help me achieve my life goals. [ 1 ] [ 2 ] [ 3 ] [ 4 ] [ 5 ]

4.Even when subjects are difficult, I still believe mastering them is worthwhile. [ 1 ] [ 2 ] [ 3 ] [ 4 ] [ 5 ]

**Part Ⅶ: Satisfaction with Life Scale (SWLS)**
*Please rate each statement based on overall life experience over the past month.*
1 = Strongly Disagree, 2 = Disagree, 3 = Slightly Disagree, 4 = Neutral, 5 = Slightly Agree, 6 = Agree, 7 = Strongly Agree

1.In most ways my life is close to my ideal. [ 1 ] [ 2 ] [ 3 ] [ 4 ] [ 5 ] [ 6 ] [ 7 ]

2.The conditions of my life are excellent. [ 1 ] [ 2 ] [ 3 ] [ 4 ] [ 5 ] [ 6 ] [ 7 ]

3.I am satisfied with my life. [ 1 ] [ 2 ] [ 3 ] [ 4 ] [ 5 ] [ 6 ] [ 7 ]

4.So far I have gotten the important things I want in life. [ 1 ] [ 2 ] [ 3 ] [ 4 ] [ 5 ] [ 6 ] [ 7 ]

5.If I could live my life over, I would change almost nothing. [ 1 ] [ 2 ] [ 3 ] [ 4 ] [ 5 ] [ 6 ] [ 7 ]

SECTION B: Data Codebook & Mapping

General Coding Notes

Status of Reverse-Coded Items (*_R):
IMPORTANT: In the provided datasets (Dataset_1 and Dataset_2), all items—including those with “_R” suffixes (i.e., CVS_COL_04_R and CVA_CTR_03_R)—are preserved in their raw response format (1 to 5).
Analysts must apply the reversal transformation formulas below prior to calculating Cronbach’s alphas, correlations, or composite scale means.

Coded by $X_{\mathrm{reversed}}=6-X_{\mathrm{original}}$ (for 5-point scales) and $X_{\mathrm{reversed}}=8-X_{\mathrm{original}}$ (for 7-point scales).

Scale scores: Calculated as the arithmetic mean of all items within each subscale after the reversal of target items.

**1. Demographic & Control Variables**

| Variable | Description/Label | Values&Coding |
| --- | --- | --- |
| P1_COUNTRY | Country/Region of study | 1 = China (Shanghai) ｜ 2 = Kazakhstan |
| P1_GENDER | Respondent’s gender | 1 = Male ｜ 2 = Female ｜ 3 = Other ｜ 9 = Prefer not to say |
| P1_AGE | Age category | 1 = 18–20 ｜ 2 = 21–23 ｜ 3 = 24–26 ｜ 4 = 27 & above |
| P1_GRADE | Academic level | 1 = Undergraduate ｜ 2 = Master’s ｜ 3 = PhD ｜ 4 = Other |
| P1_PAREDU | Parental highest education | 1 = Junior high or below ｜ 2 = Senior high/vocational ｜ 3 = Bachelor’s ｜ 4 = Master’s or above ｜ 5 = Not sure |

**2. Psychometric Scale Mapping**

| Latent Variable (Mean Score) | Mapping to Section A | Number of Items | Scoring Notes & Item Names |
| --- | --- | --- | --- |
| PALS_MAS_MEAN | Part Ⅱ (Mastery Climate) | 4 | Forward (PALS_MAS_01 to 04) |
| PALS_PER_MEAN | Part Ⅱ (Performance Climate) | 4 | Forward (PALS_PER_01 to 04) |
| PALS_PAV_MEAN | Part Ⅱ (Performance-Avoidance) | 3 | Forward (PALS_PAV_01 to 03) |
| PPASS_MEAN | Part Ⅲ (PPASS Support) | 5 | Forward (PPASS_01 to 05) |
| AEQ_ENJ_MEAN | Part Ⅳ (Enjoyment) | 3 | Forward (AEQ_ENJ_01 to 03) |
| AEQ_HOP_MEAN | Part Ⅳ (Hope) | 3 | Forward (AEQ_HOP_01 to 03) |
| AEQ_PRI_MEAN | Part Ⅳ (Pride) | 3 | Forward (AEQ_PRI_01 to 03) |
| AEQ_ANX_MEAN | Part Ⅳ (Anxiety) | 4 | Forward (AEQ_ANX_01 to 04) |
| AEQ_SHA_MEAN | Part Ⅳ (Shame) | 3 | Forward (AEQ_SHA_01 to 03) |
| AEQ_BOR_MEAN | Part Ⅳ (Boredom) | 3 | Forward (AEQ_BOR_01 to 03) |
| AEQ_ANG_MEAN | Part Ⅳ (Anger) | 3 | Forward (AEQ_ANG_01 to 03) |
| AEQ_POS_MEAN | Part Ⅳ (Positive Emotions) | 9 | Mean of ENJ, HOP, and PRI scores |
| AEQ_NEG_MEAN | Part Ⅳ (Negative Emotions) | 13 | Mean of ANX, SHA, BOR, and ANG scores |
| CVS_COL_MEAN | Part Ⅴ (Collectivism) | 4 | Item 4 is Reverse-scored (CVS_COL_04_R) |
| CVS_PWD_MEAN | Part Ⅴ (Power Distance) | 3 | Forward (CVS_PWD_01 to 03) |
| CVS_UAV_MEAN | Part Ⅴ (Uncertainty Avoidance) | 2 | Forward (CVS_UAV_01 to 02) |
| CVA_CTR_MEAN | Part Ⅵ (Perceived Control) | 4 | Item 3 is Reverse-scored (CVA_CTR_03_R) |
| CVA_VAL_MEAN | Part Ⅵ (Perceived Value) | 4 | Forward (CVA_VAL_01 to 04) |
| SWLS_MEAN | Part Ⅶ (Life Satisfaction) | 5 | Forward (SWLS_01 to 05), 7-point scale |
